# The *WtmsDW* Locus on Wheat Chromosome 2B Controls Major Natural Variation for Floret Sterility Responses to Heat Stress at Booting Stage

**DOI:** 10.3389/fpls.2021.635397

**Published:** 2021-03-29

**Authors:** Million F. Erena, Iman Lohraseb, Isabel Munoz-Santa, Julian D. Taylor, Livinus C. Emebiri, Nicholas C. Collins

**Affiliations:** ^1^School of Agriculture Food and Wine, The University of Adelaide, Adelaide, SA, Australia; ^2^Department of Statistics and Operations Research, University of Valencia, Valencia, Spain; ^3^Graham Centre for Agricultural Innovation, Charles Sturt University, Wagga Wagga, NSW, Australia; ^4^New South Wales Department of Primary Industries, Wagga Wagga, NSW, Australia

**Keywords:** wheat, heat tolerance, male sterility, floret sterility, auricle distance, QTL

## Abstract

Heat stress at booting stage causes significant losses to floret fertility (grain set) and hence yield in wheat (*Triticum aestivum* L.); however, there is a lack of well-characterized sources of tolerance to this type of stress. Here, we describe the genetic analysis of booting stage heat tolerance in a cross between the Australian cultivars Drysdale (intolerant) and Waagan (tolerant), leading to the definition of a major-effect tolerance locus on the short arm of chromosome 2B, *Wheat thermosensitive male sterile Drysdale/Waagan* (*WtmsDW*). *WtmsDW* offsets between 44 and 65% of the losses in grain set due to heat, suggesting that it offers significant value for marker-assisted tolerance breeding. In lines lacking the *WtmsDW* tolerance allele, peaks in sensitivity were defined with reference to auricle distance, for various floret positions along the spike. Other (relatively minor) floret fertility response effects, including at the *Rht-D1* dwarfing locus, were considered likely escape artifacts, due to their association with height and flowering time effects that might interfere with correct staging of stems for heat treatment. Heat stress increased grain set at distal floret positions in spikelets located at the top of the spike and increased the size of spikelets at the base of the spike, but these effects were offset by greater reductions in grain set at other floret positions. Potentially orthologous loci on chromosomes 1A and 1B were identified for heat response of flowering time. The potential significance of these findings for tolerance breeding and further tolerance screening is discussed.

## Introduction

Heat stress reduces yields of wheat in most global production environments, and the situation is worsening with climate change (Asseng et al., 2015).

Elevated temperatures accelerate development, senescence, and water use, reducing the opportunity to accumulate biomass and therefore yield (Asseng et al., 2011; Hunt et al., 2018). Heat waves (> 30°C for one to several days) during sensitive reproductive development stages also impact directly on grain set (floret fertility) and grain development. Wheat has two periods of sensitivity to the floret sterility effects: during booting (flag leaf sheath extending, 1–2 weeks before anthesis), and 2–3 days before anthesis (Saini and Aspinall, 1982; Tashiro and Wardlaw, 1990a; Craufurd et al., 2013; Prasad and Djanaguiraman, 2014; Barber et al., 2017). Heat stress during early grain filling reduces the weight of individual grains (Tashiro and Wardlaw, 1990b; Stone and Nicolas, 1995). Grain filling heat stress can also affect grain physical and biochemical traits determining processing characteristics and end-use quality (Stone and Nicolas, 1998; Wrigley, 2007).

Modeling has indicated that every further degree Celsius rise in mean global temperature would result in a 6% loss in wheat yields worldwide (Asseng et al., 2015). In Australia, it was estimated that heat shocks during reproductive development reduced grain number and individual grain weight by 3.6 and 18.1%, respectively, translating to a yield loss of 20.8%, in the mid-maturing wheat cultivar Janz, over the period 1985–2017 (Ababaei and Chenu, 2020).

To help limit these yield losses, tolerant wheat cultivars able to withstand heat waves during reproductive development could be grown. However, breeder’s efforts to identify heat tolerance in the field are hampered by the unpredictable timing and intensity of natural heat events and the narrow developmental windows of sensitivity. Selection using molecular markers would be more convenient than phenotypic selection. However, this strategy would firstly require identification of loci controlling major variation for heat tolerance.

Various efforts have been made to identify grain filling heat tolerance quantitative trait loci (QTL) in hexaploid wheat, involving transfers of potted plants into a growth chamber set at high temperatures, at 7–10 days after anthesis (Mohammadi et al., 2008; Mason et al., 2010, 2011; Shirdelmoghanloo et al., 2016; Guo et al., 2020; Lu et al., 2020). However, relatively few studies have targeted floret fertility responses to heat stress applied at booting or at around anthesis (Barber et al., 2017). Barber et al. (2017) identified one weak tolerance QTL for booting stage, plus two tolerance QTL for anthesis stage that were associated with a locus for dwarfing (*Rht-D1*) and flowering time (*Ppd-D1*), respectively.

Accordingly, in the current study, we performed QTL analysis on floret fertility responses to heat stress applied at booting, in a cross between the Australian wheat cultivars Drysdale and Waagan. An initial study indicated Drysdale was relatively sensitive to heat stress at this stage and Waagan tolerant (Erena, 2018). We have also used this population to identify grain filling heat tolerance QTL (Shirdelmoghanloo et al., 2016), providing the opportunity to compare tolerance QTL for the two developmental stages.

## Materials and Methods

### Plant Genetic Materials and Markers

An initial experiment was undertaken to discover QTL for heat responsiveness of traits in a Drysdale × Waagan F_1_-derived doubled haploid (DH) population, by applying a brief heat stress at booting stage (“DH QTL experiment”). The population of 144 lines, and the genetic map of 551 nonredundant marker loci, has previously been described (Shirdelmoghanloo et al., 2016).

After identifying a strong floret fertility heat tolerance locus on chromosome 2B (*WtmsDW*), KASP^TM^ assays were utilized to map this region in more detail and develop *WtmsDW* near-isogenic lines. KASP^TM^ assays were run using an automated SNPLine system and Kraken^TM^ software (DNA LGC Limited, London, United Kingdom). In addition to using a pre-existing KASP assay for the *Ppd-B1* gene, three other KASP markers in the region were developed, including one based on the SNP *wsnp_JD_c3732_4781170* from the wheat 9k Illumina iSelect SNP array (Cavanagh et al., 2013) and *AHW_DW_001* and *AHW_DW_014* based on SNPs between Drysdale and Chinese Spring identified using the DAWN genomics tool (Watson-Haigh et al., 2018; Supplementary Table 1). KASP markers were scored on the DH lines to confirm their locations (Supplementary Figure 1).

A Drysdale × Waagan recombinant inbred line (RIL) population was made for developing *WtmsDW* near-isogenic lines. The RIL population was derived by single-seed descent from F_2_ plants of a Drysdale × Waagan cross. The four KASP markers were used to identify an F_6_ RIL plant that was heterozygous for the *WtmsDW* region. This was then used to derive a *WtmsDW*-heterozygous F_8_ plant by two further rounds of single-seed descent with marker selection. The progeny of this single plant were screened to identify three plants homozygous for each allele type. These were then allowed to self-pollinate to establish seed stocks of the six near-isogenic lines (NILs): NIL-T-1, NIL-T-2, and NIL-T-3 (Waagan allele, tolerant) and NIL-I-1, NIL-I-2, and NIL-I-3 (Drysdale allele, intolerant). Four progeny of the same *WtmsDW*-heterozygous F_8_ plant (sibs of the selected NILs), together with the Drysdale and Waagan parents, had been subjected to genomic profiling using DArTSeq^TM^. These data were used to identify the chromosome segments segregating in this material and hence differing between the NILs. Genomic locations of the DArTSeq markers in the wheat IWGSC v1.0, Chinese Spring reference genome sequence had been determined using BLAST searches.

### Greenhouse Conditions, Heat Treatment, and Data Collection

To phenotype the Drysdale × Waagan DH population in the DH QTL experiment, plants were grown in the Plant Accelerator facility at the Waite Campus of the University of Adelaide, using procedures similar to Shirdelmoghanloo et al. (2016). Plants were sown on 16th March 2014 and grown in a naturally lit evaporatively cooled greenhouse compartment, where max-day/min-night temperatures averaged 20/17°C and day/night relative humidity averaged 68/76% throughout growth. The greenhouse temperature reached 27.2°C once during the treatment period due to high outside temperatures. The experiment was arranged in three sections of the greenhouse. Each section comprised a rectangular array of pots, each of them indexed by its row and column. The experiment was designed in a split plot layout with four blocks (replicates). Genotypes (DH lines and parents) were randomly allocated to main plots comprising pairs of adjacent pots in rows and treatments (control and heat) to subplots comprising the two pots within main plots. Each plant was heat treated when the main stem reached a certain growth stage, defined by the distance between the auricles (collars) of the flag leaf and the next leaf down (auricle distance, AD; Jagadish et al., 2014). Treatments began on the day the AD on the main stem was closest to 3 cm (for replicates 1 and 3) or 9 cm (for replicates 2 and 4), i.e., during mid-booting (growth stages Z41–Z46; Zadoks et al., 1974). AD was also measured to the nearest 0.5 cm on the day of heat treatment, on both the main stem and the most advanced tiller, and these two stems were marked with different color tags. Plants were moved to a walk-in growth chamber (Conviron BDW120) set at 14 h day-length and 37/27°C day/night temperature. Maximum temperature was held for 8 h, with 3-h ramping periods either side (Supplementary Figure 2A). Day/night relative humidity was around 60/80% in the chamber. While in the chamber, pots sat in trays containing ∼2 cm of water to minimize drought stress. Plant movement in and out of the chambers each day was done at the start of the night cycle. After 3 days, plants were moved back to the greenhouse to complete their development. Traits (Table 1) were scored on the tagged stems. The AD of the tagged advanced tiller averaged 1.6 and 6.5 cm when the main stem AD was 3 and 9 cm, respectively. Thus, four developmental stages of the measured stems were defined for analysis of heat responses: 1.6, 3, 6.5 and 9 cm AD.

**TABLE 1:** Traits measured in the DH QTL experiment.

Trait abbreviation	Description
Day.AD	No. days from sowing to targeted auricle distance (pre-heat)
Day.Anth	No. days from sowing to first anthesis (extrusion of first anther) on the main stem
Day.ADtoAnth	Trait “Day.Anth” minus trait “Day.AD”
AwnEm.PreH	Length of emerged awns at targeted auricle distance (pre-heat; cm)
AD.Mat	Auricle distance at maturity (cm)
Ht.Mat	Plant height at maturity, soil level to bottom of spike (cm)
SpkL.Mat	Spike length at maturity, to glumes of terminal spikelet (cm)
AwnL.Mat	Length of awns above terminal spikelet at maturity (cm)
UndvSplt.Spk	No. of basal under-developed spikelets per spike at maturity. Under-developed spikelets were defined as those with awn length < 50% that of the spikelets from the middle of the spike.
NoSplt.Spk	Total spikelet no. per spike at maturity
GrNoSplt.1&2.Top	Grains per developed spikelet, floret positions 1 and 2, top third of spike
GrNoSplt.1&2.Mid	Grains per developed spikelet, floret positions 1 and 2, middle third of spike
GrNoSplt.1&2.Bot	Grains per developed spikelet, floret positions 1 and 2, bottom third of spike
GrNoSplt.>2.Top	Grains per developed spikelet, floret positions > 2, top third of spike
GrNoSplt.>2.Mid	Grains per developed spikelet, floret positions > 2, middle third of spike
GrNoSplt.>2.Bot	Grains per developed spikelet, floret positions > 2, bottom third of spike
GrNoSplt.Spk	Grain number per developed spikelet, across all floret and spike positions

*Day.AD, Day.Anth, and Day.ADtoAnth were only scored in main stems.*

Transfer of the Drysdale × Waagan DH lines to the growth chamber involved a change in day length (10.3 h in the greenhouse vs. 14 h in the growth chamber) as well as temperature. To clarify which environmental factor was relevant to *WtmsDW*, a second experiment was performed using the *WtmsDW* NILs (“*WtmsDW* validation experiment”). Plants were sown on 22nd July 2020 in the greenhouse, with a natural day length of 11.5 h at booting. Reach-in chambers (Conviron PGC20) containing a mixture of halogen incandescent lamps and fluorescent tubes were used to apply four treatments in which heat stress and day length were varied (Supplementary Figures 2A–D). As only two chambers were available, the earliest developing plants were used for the two treatments involving heat, and the slightly later plants were then used for the two treatments involving no heat. For the former, the main stem was tagged and used for data collection, while in the latter, either a main stem or advanced tiller was used. Each plant was treated when AD on the tagged stem was 6 cm. Treatment duration was 2 or 3 days, for treatments with and without heat stress, respectively. After treatment, plants were moved back to the greenhouse to complete their development. Plant arrangement in the greenhouse was a completely randomized design, with 19 to 41 plants (replicates) ultimately being treated per allele/treatment combination.

### Phenotypic Modeling for DH QTL Experiment

Each trait in each stem was analyzed using the following linear mixed model:

(1) $$\text{y}=\text{X}\,\tau +{\text{Z}}_{g}\,\text{g}+{\text{Z}}_{b}\,{\text{u}}_{b}+{\text{Z}}_{m}\,{\text{u}}_{m}+\text{e}$$

where ***y*** is the vector of observations, ***τ*** is the vector of fixed effects containing the terms to capture the treatment by genotype effects (DH or check lines) with associated design matrix ***X***, **g** is the vector of random genetic effects with design matrix **Z**_*g*_, **u**_*b*_ is the vector of random block effects with design matrix **Z**_*b*_, **u**_*m*_ is the vector of random main plot effects with design matrix **Z**_*m*_, and **e** is the residual error.

The joint distribution of (**g**,**u**_*b*_,**u**_*m*_,**e**) was assumed to be Gaussian with zero mean and variance covariance matrix:

$$\left[\begin{array}{cccc} {\text{G}}_{g}\,\left(\gamma_{g}\right) & 0 & 0 & 0\\ 0 & \sigma_{b}^{2}\,{\text{I}}_{b} & 0 & 0\\ 0 & 0 & \sigma_{m}^{2}\,{\text{I}}_{m} & 0\\ 0 & 0 & 0 & \text{R}\,\left(\phi \right) \end{array}\right]$$

where $\gamma_{g},\sigma_{b}^{2},\sigma_{m}^{2}$, and ϕ are unknown variance parameters associated with the genetic effects, block variance, main plot variance, and residual error, respectively. The **I** matrix denotes the identity matrix. Pots in the greenhouse were divided in three sections so **R**(ϕ) was assumed to be the direct sum of ${⊕}_{s=1}^{3}\sigma_{s}^{2}\,\Sigma \,\left(\rho_{c\,s}\right)⊗\Sigma \,\left(\rho_{r\,s}\right)$. The parameter $\sigma_{s}^{2}$ denotes the error variance and **Σ**(ρ_*rs*_)⊗**Σ**(ρ_*cs*_) refers to an autoregressive process of order one in the column and row directions in section *s.* This is a plausible model to account for the correlation between errors due to the neighboring pots within each section. Most importantly, **G**_*g*_(γ_*g*_) represents the variance covariance matrix of the genetic effects for the DH lines only. There were three treatments so **g** was partitioned into **g**_*c*_,**g**_3_, and **g**_9_ for the control, heat applied at the AD closest to 3 and 9 cm genetic effects, respectively. The variance matrix **G**_*g*_(γ_*g*_) was then assumed to be:

$$\left[\begin{array}{ccc} \sigma_{c}^{2} & \sigma_{c\,3} & \sigma_{c\,9}\\ \sigma_{c\,3} & \sigma_{3}^{2} & \sigma_{39}\\ \sigma_{c\,9} & \sigma_{39} & \sigma_{9}^{2} \end{array}\right]⊗{\text{I}}_{g}$$

For all traits measured, tolerance genetic effects at the targeted development stages were derived based on the conditional distribution of the heat genetic effects given the control genetic effects (Lemerle et al., 2006). For instance, the tolerance genetic effects obtained from the application of the heat treatment at the AD closest to 3 cm, **g**_*t*3_, is distributed as follows:

(2) $${\text{g}}_{t\,3}={\text{g}}_{3}|{\text{g}}_{c}∼N\left(E\left({\text{g}}_{3}-\beta_{3}{\text{g}}_{c}\right)=0,\sigma_{t\,3}^{2}{\text{I}}_{g}\right)$$

where $\beta_{3}=\rho_{c\,3}\,\frac{\sigma_{3}}{\sigma_{c}}$, ρ_*c*3_ = $\sigma_{c\,3}/{\sqrt{(\sigma }}_{c}^{2}\sigma_{3}^{2})$ and σt⁢32=σ32(1-ρ)c⁢32.

Following (2), **g**_*t*3_ can be viewed as residuals from a random regression of **g**_3_ against **g**_*c*_ with intercept zero and slope β_3_. Genotypes with large positive residuals have higher tolerance to heat stress than an average genotype while large negative residuals suggest poor tolerance. A similar derivation can be used to obtain tolerance effects for the application of the heat treatment at the AD closest to 9 cm, i.e., **g**_*t*9_.

Benefits of defining tolerance effects in this way are that the effects are reported in the original unit of measurement and are independent of the control genetic effects. This avoids the problem inherent in some other commonly used response indexes, such as the Heat Susceptibility Index (Mason et al., 2010) that tend to be influenced by control *per se* performance. Tolerance effects defined in this way were also used by McDonald et al. (2015) and Mahjourimajd et al. (2016).

All models (1) were diagnostically assessed to ensure that the assumptions of normality and homoscedasticity of errors were satisfied and, where appropriate, traits were transformed. The methods described in Gilmour et al. (1997) were used to account for possible spatial trends across the experimental layout in the glasshouse. Where appropriate, the linear mixed model (1) was adapted to include linear row and column terms in the fixed part of the model or random row or column effects. The significance of the correlations of the error section terms was also assessed.

The method of residual maximum likelihood (REML) was used for variance parameter estimation (Patterson and Thompson, 1971). The best linear unbiased predictions (eBLUPs) for each line at each treatment were extracted from the model, and the tolerance effects were derived as residuals from the random regressions using the REML estimates. All analyses were conducted in the R environment (R Core Team, 2020) using the ASReml-R software (Butler et al., 2017).

### QTL Mapping

For each of the traits, the eBLUPs for the DH lines under control and the two different heat conditions, as well as the eBLUPs for heat tolerance calculated from (2), were used to conduct QTL analysis. QTL mapping was performed using the approach of Shirdelmoghanloo et al. (2016). Following simple interval mapping, candidate QTL were used as co-factors for composite interval mapping (CIM), setting the minimum co-factor proximity to 30 cM and maximum step size to 10 cM. Putative QTL were considered significant if they exceeded a genome-wide LOD threshold of 1.8 calculated using the adjusted Bonferroni-corrected *p* value with significance level α=0.05 (Li and Ji, 2005). To assist interpretation, QTL effects linked within ∼30 cM were grouped to the same numbered QTL locus. All QTL analyses were conducted using the statistical computing environment GenStat version 16 (Payne, 2009; VSN International, 2020). Markers in four genomic locations showed segregation distortion (on linkage groups 2B1, 3B1, 5A2, and 6B2; χ^2^ test, *p* < 0.01), but these were not located at any of the QTL reported in the current study.

### Relationship of Treatment Stage to Heat-Induced Sterility and Its Interaction With Stem Type, Floret Position, and Genotype at WtmsDW and Rht Loci

The markers most closely associated with *WtmsDW* effects (*Ppd-B1*, *wsnp_Ex_c5412_9565527*, *wsnp_JD_c3732_4781170*, *wsnp_RFL_Contig4483_5312236*) were used to infer the *WtmsDW* allele (Drysdale or Waagan) carried by each DH line (marker recombinants being excluded). *Rht-B1* and *Rht-D1* alleles carried by DH lines were known from scores of diagnostic KASP markers for these genes (Shirdelmoghanloo et al., 2016). Fertility was plotted against AD length on the day of treatment and 3rd-order polynomial trend curves fitted using Microsoft Excel.

### Relationships to Other Fertility Loci

Other fertility loci responsive to temperature and other environmental factors were considered for their potential relationships to *WtmsDW*. These included loci described in wheat (Kuchel et al., 2007; Mason et al., 2010, 2011, 2013; Pinto et al., 2010; Zheng et al., 2010; Pandey et al., 2014; Sharma et al., 2016; Shirdelmoghanloo et al., 2016; Barber et al., 2017; Bhusal et al., 2017; Pradhan et al., 2019; Guo et al., 2020; Lu et al., 2020; Selva et al., 2020 and references therein), durum (*Triticum durum* Desf.) (El Hassouni et al., 2019), barley (*Hordeum vulgare* L.) (Romagosa et al., 1999; Malosetti et al., 2004), and rice (*Oryza sativa* L.) (Yu et al., 2017; Zhu et al., 2017; Fan and Zhang, 2018 and references therein; Khlaimongkhon et al., 2019; Cao et al., 2020; Nubankoh et al., 2020). Marker sequences were accessed from Cavanagh et al. (2013), GrainGenes^1^, Gramene^2^, NCBI^3^, and Diversity Arrays Technology^4^. Marker sequences were located in the wheat IWGSC v1.0, Chinese Spring reference genome sequence using an in house BLAST tool, or in the rice Nipponbare IRGSP Reference sequence 1.0 by BLAST search at the Rice Genome Program site^5^. To establish further wheat-rice genomic interval relationships, gene sequences were accessed through the Rice Genome Program site and the DAWN tool, and homologues located in the respective genomes by BLAST search.

## Results

### Trait Responses

**FIGURE 1:**
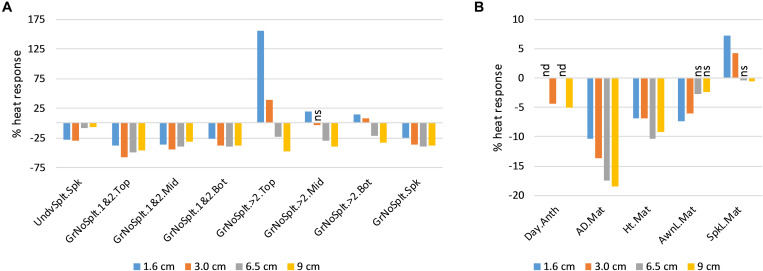
Average trait heat responses (%) of Drysdale × Waagan DH lines, relative to controls. All responses were significant at *p* < 0.01 unless indicated. nd, not determined; ns, not significant. **(A)** Fertility-related traits. **(B)** Growth and development traits. See Table 1 for trait key.

In the DH QTL experiment, all traits measured after heat treatment responded to heat in the DH lines, except for number of spikelets per spike (Figure 1). Heat decreased the number of spikelets at the bottom of the spike that were classified as underdeveloped (UndvSplt.Spk), by up to 28%. Heat consistently decreased grain set in the lowest two floret positions in the spikelets (GrNoSplt.1&2), but it increased grain set in the upper floret positions (GrNoSplt.>2) for stems treated at the earlier developmental stages (1.6 and 3 cm AD). However, the overall effect of heat at these early stages on grain set (GrNoSplt.Spk; Figure 1A) was still negative because the > 2 positions produced far fewer seeds than the lower two floret positions (e.g., 0.15 vs. 0.94 grains per spikelet, in the top third of the spike, under heat). Heat accelerated the time from sowing to first anthesis (Day.Anth), by up to 5% (3.1 days). It increased final spike length (SpkL.Mat) in stems exposed to heat at the earlier stages, by up to 7.3%, but decreased it by 0.6% for the stems exposed at the latest stage. This was due to responses in rachis internode length because total spikelet number per spike was unaffected. Heat decreased auricle distance and height at maturity (AD.Mat and Ht.Mat), with the effects being the greatest for the stems exposed to heat at later stages. Heat decreased awn length at maturity (AwnL.Mat), with the effects being the greatest for the stems exposed to heat at the earlier stages (Figure 1B).

### Quantitative Trait Loci

Overall, 221 QTL effects were defined for *per se* traits (in control or heat) and 54 QTL effects defined for heat tolerance effects (responses) (Supplementary Table 1). The QTL effects were grouped into 33 genomic locations (*QTLx* designations). Of these, 16 coincided with loci previously identified in this population (Shirdelmoghanloo et al., 2016), while 17 were new (*QTL30* to *QTL45*). As previously reported, this population segregated for major height effects at the *Rht-D1* and *Rht-B1* loci but was relatively uniform for flowering time (largest effect 2.9 days at *QTL18* on chromosome 4B).

Six loci were defined for heat responses of floret fertility (Table 2). *QTL36* on the short arm of chromosome 2B had the strongest tolerance effect, controlling up to 43% of the variation, with tolerance deriving from the tolerant parent Waagan. Its *per se* fertility effects were almost exclusively observed under heat. It was also the most consistently expressed fertility response locus, showing an effect for 18 out of the 24 tested floret position and treatment-stage combinations (Supplementary Table 2). *QTL36* also showed an effect for awn length *per se*. *QTL36* effects were found to arise from sterility in the male reproductive organs (data not shown). Accordingly, we named this major *QTL36* heat tolerance locus *wheat thermosensitive male sterile Drysdale/Waagan* (*WtmsDW*). Additional greenhouse experiments to characterize *WtmsDW* effects on pollen and anther development will be described in a separate paper.

**TABLE 2:** Loci for heat responses of floret fertility identified in the DH QTL experiment.

Locus	Linkage group	Position (cM)	Peak markers	Tolerance allele^*a*^	LOD	*R*^2^	Additive effect
*QTL32*	1B	66.1	*wsnp_Ex_c58292_59652859*	D	3.8	8.6	0.01
*QTL36^*b*^ (WtmsDW)*	2B1	74.3–84.8	*Ppd-B1*; *wsnp_JD_c3732_4781170*; *wsnp_RFL_Contig4483_5312236*	W	18	43	0.37
*QTL39*^*b*^	3B2	80.9–105.9	*wsnp_Ex_c9594_15882022*; *wsnp_Ex_rep_c101457_86818160*	W	5.0	15	0.11
*QTL18*	4B	127.5	*wsnp_Ku_c11570_18860306*	D	4.3	9.3	0.01
*QTL19^*b*^ (Rht-D1)*	4D	0.0–2.9	*Rht-D1*; *wsnp_CAP11_c356_280910*	D or W	6.8	12	0.18
*QTL43*^*b*^	7A2	43.2–67.0	*wsnp_Ex_c2268_4251636*; *wsnp_Ex_c12102_19361467*	W	4.0	6.6	0.14

*^*a*^*D*, Drysdale; *W*, Waagan.*

*^*b*^Floret fertility tolerance effects were observed at these loci for multiple floret type/treatment timing combinations; figures in the last three columns are for the strongest effect.*

The other five loci for floret fertility response (*QTL18*, *19*, *32*, *39* and *43*) were weaker and less consistently expressed than *WtmsDW* (Table 2 and Supplementary Table 2). They were associated with various developmental traits (Supplementary Table 2), suggesting these fertility effects may have been developmental artifacts rather than due to genuine tolerance (see section “Discussion”).

For other (non floret fertility) traits, heat-response effects were observed at 13 loci (Table 3).

**TABLE 3:** Heat-response QTL effects for traits other than floret fertility identified in the DH QTL experiment.

Locus	Trait	Linkage group	Position (cM)	Tolerance allele^*a*^	Treatment stage (AD)^*b*^	LOD	*R*^2^	Additive effect
*QTL30*	Day.Anth	1A1	97.0	W	3	3.5	10.0	0.16
*QTL33*	Day.Anth	1B	135.2	D	9	3.5	8.9	0.74
*QTL29*	Day.ADtoAnth	7B	56.9	D	3	6.9	18.8	0.79
*QTL15*	AD.Mat	4A2	41.6	D	1.6 and 9	4.3	1.5	0.01
*QTL15*	Ht.Mat	4A2	41.6	D	1.6	4.1	2.1	0.01
*QTL17 (Rht-B1)*	AD.Mat	4B	83.9	W	9	47.0	44.5	0.01
*QTL17 (Rht-B1)*	Ht.Mat	4B	83.9	W	1.6	37.5	41.2	0.01
*QTL18*	AD.Mat	4B	141.3	W	9	4.9	1.8	0.01
*QTL19 (Rht-D1)*	AD.Mat	4D	0.0	D	9	46.0	41.9	0.01
*QTL19 (Rht-D1)*	Ht.Mat	4D	0.0	D	1.6	39.3	44.8	0.01
*QTL28*	AD.Mat	7A2	33.7	W	9	5.8	3.0	0.01
*QTL34*	Ht.Mat	2A	147.0–160.2	D	3 and 6.5	4.3	12.1	1.56
*QTL29*	AwnL.Mat	7B	45.8	D	6.5	3.7	9.4	0.05
*QTL41*	AwnL.Mat	5D2	40.2	W	3	3.5	11.3	0.01
*QTL9*	SpkL.Mat	2D4	7.6	D	1.6	3.6	9.2	0.01
*QTL17 (Rht-B1)*	UndvSplt.Spk	4B	83.9	D	3 and 9	16.9	26.3	0.01
*QTL18*	UndvSplt.Spk	4B	135.5	D	3 and 9	8.7	11.6	0.01
*QTL19 (Rht-D1)*	UndvSplt.Spk	4D	0.0	W	3 and 9	10.5	13.4	0.01
*QTL25*	UndvSplt.Spk	6A	74.7	D	3 and 9	4.1	4.5	0.07
*QTL5.2*	UndvSplt.Spk	2A	93.6	D	1.6 and 6.5	3.7	9.6	0.09

*Effects are grouped by trait type: anthesis-related, height-related, awn or spike length-related, and underdeveloped lower spikelets. See Table 1 for trait key.*

*^*a*^*W*, Waagan; *D*, Drysdale.*

*^*b*^Treatments giving QTL effects, based on auricle distance (AD, cm) at time of heat treatment. Where QTL effects were detected at two treatment times, figures in the last three columns are for the strongest effect.*

### Interactions of WtmsDW Heat Tolerance Expression With Treatment Stage, Stem Type, Floret Position, and Rht Genotype

Sterility in main stems and tillers of DH mapping lines showed similar response curves, to heat applied at the various stem developmental stages, either in the lines carrying the Drysdale or Waagan alleles at *WtmsDW* (Supplementary Figure 3). Therefore, data from main stem and tillers were combined for subsequent analysis.

**FIGURE 2:**
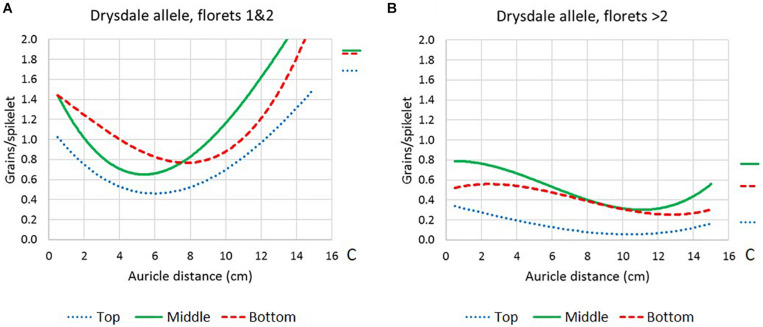
Floret fertility response curves (polynomial regression, order-3) in Drysdale × Waagan DH lines that had been heat treated at different stem developmental stages as defined by auricle distance, in the top, middle, and bottom third of the spike. Only lines carrying the Drysdale (intolerance) allele at *WtmsDW* are represented. **(A)** Floret positions 1 and 2 in the spikelets. **(B)** Floret positions > 2 in the spikelets. Average fertility in control plants C are represented by the lines to the right of the plots.

Doubled haploid lines carrying different allele combinations at the *Rht-B1* and *Rht-D1* height loci were also compared (Supplementary Figure 4). The two semi-dwarf types and the talls showed similar response patterns, while intolerance in the double-dwarf types peaked at a shorter AD. Therefore, the double-dwarfs were excluded from the analysis to compare floret positions (Figure 2 and Supplementary Figure 5).

In the lowest two floret positions in the spikelets (positions 1 and 2), intolerance peaked at AD ∼5.5 cm in the middle of the spike, while at the top and bottom of the spike, intolerance peaked later, at around 6 and 8 cm, respectively (Figure 2A). Floret positions > 2 in the spikelets peaked in intolerance at approximately 11, 11, and 12.5 cm AD, in the top, middle, and bottom of the spike, respectively (Figure 2B).

The DH lines carrying the Waagan *WtmsDW* allele maintained high levels of fertility across all stages where heat stress was applied (Supplementary Figures 5C,D). Thus, it was unlikely that *WtmsDW* exerted its fertility effects by merely altering the AD vs. spike developmental stage relationship, resulting in escape.

### Further Mapping in the WtmsDW Region and Relationships to Other Fertility Loci

**FIGURE 3:**
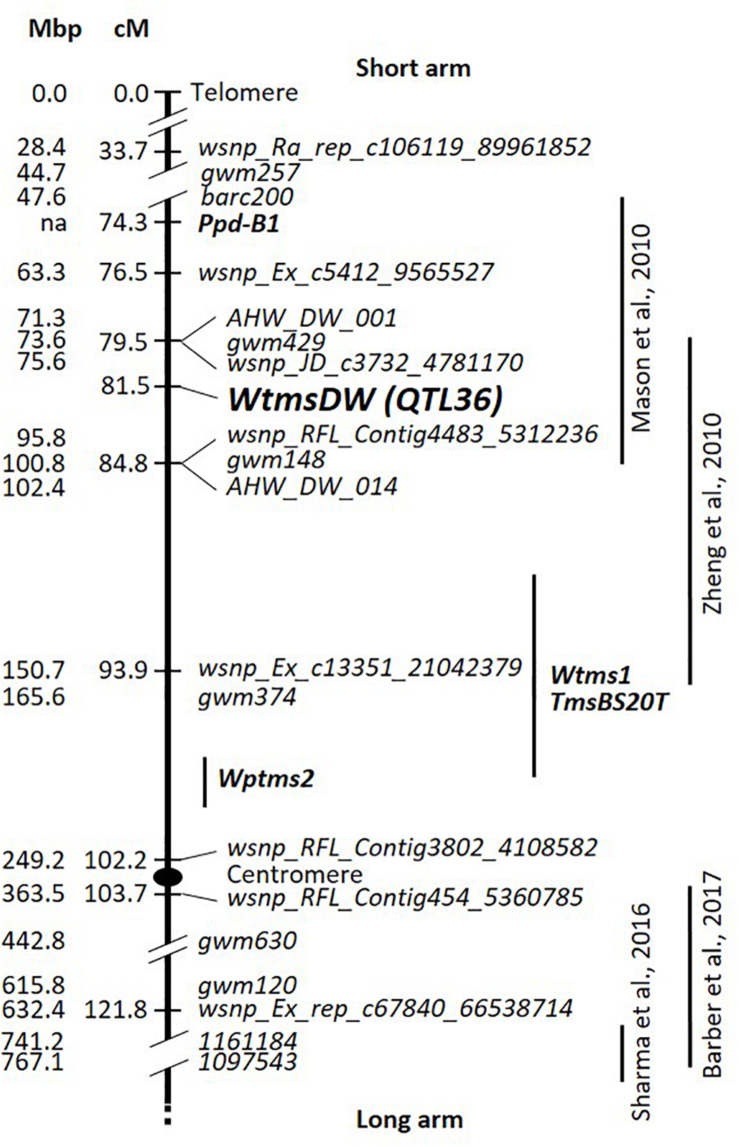
Drysdale × Waagan DH genetic map predominantly for the short arm of wheat chromosome 2B. Mbp locations of markers in the IWGSC v1.0, Chinese Spring reference genome sequence are shown to the left. The centromere position is from Alonge et al. (2020). Positions of temperature-responsive fertility QTL from four other studies are shown on the right. Temperature and/or photoperiod-responsive male-sterility loci *Wtms1*, *TmsBS20T*, and *Wptms2* studied for hybrid breeding research are also shown (Xing et al., 2003; Guo et al., 2006a; Ru et al., 2015). Positions of the loci from other studies were located approximately, based on position of markers from the respective studies in the genomic sequence.

Based on DH lines that were nonrecombinant for markers spanning the *WtmsDW* region (33.7–93.9 cM), heat tolerance genetic effects proved to be a good predictor of *WtmsDW* allele type. For 68 out of the 81 nonrecombinants, positive and negative values correctly indicated the presence of the Waagan and Drysdale alleles, respectively. Inconsistencies were not clearly associated with any particular *Rht-B1*/*Rht-D1* genotype combination (Supplementary Figure 1). Accordingly, *WtmsDW* genotype was inferred for the remaining (recombinant) DH lines, which then allowed *WtmsDW* to be mapped as a single point locus. It was located to a 5.3-cM marker interval (Figure 3), corresponding to the physical interval 76.82 to 95.76 Mb on chromosome 2B in the IWGSC v1.0, Chinese Spring reference genome sequence (Supplementary Figure 1).

The DH line WW28450 carried a spontaneous deletion on chromosome 2B from 104.24 Mb upwards (Supplementary Figure 1), essentially covering the whole of the short arm, including the *WtmsDW* locus (Figure 3). This line was phenotypically intolerant to heat stress but fully fertile under control conditions (Supplementary Figure 1).

Previously described loci on wheat chromosome 2B influencing fertility responses to environmental factors were considered for their potential relationships to *WtmsDW*. Mason et al. (2010) reported a QTL for response (Heat Susceptibility Index (HSI)) of kernel number per main spike to post-anthesis heat stress, between markers *gwm148* and *barc200*, which is in the vicinity of *WtmsDW* (Figure 3). Zheng et al. (2010) described interactions of kernel number per square meter with several environmental covariates including cumulative degree-days during the 6 days around meiosis, associated with the markers *gwm429* and *gwm374*, which is also close to *WtmsDW* (Figure 3). Barber et al. (2017) reported a relatively weak QTL affecting interaction of heat stress during early booting with floret fertility, associated with the marker *gwm120*. However, *gwm120* is ∼40 cM from *WtmsDW* on the long arm of chromosome 2B (Figure 3). Sharma et al. (2016) described a HSI effect of grain number per spike, associated with markers *1161184* and *1097543*, calculated by comparing late vs. timely sown field trials. However, these markers are > 40 cM from *WtmsDW* on the long arm (Figure 3).

The *Wtms1*, *TmsBS20T*, and *Wptms2* male sterility loci on chromosome 2B have been defined in the context of hybrid breeding research. The *Wtms1* and *TmsBS20T* loci are expressed if it is colder than 10°C during spike development. These loci have been mapped 4.8 and 4.5 cM from marker *gwm374*, respectively (Xing et al., 2003; Ru et al., 2015), which places them at least ∼10 cM from *WtmsDW* (Figure 3). The *Wptms2* locus expressed sterility in late sowings (Guo et al., 2006a,b), thus requiring long days and/or high temperature for expression. It was mapped 6.9 cM below *gwm374* (Guo et al., 2006a), placing it ∼20 cM from *WtmsDW* (Figure 3).

In rice, BLAST searches with gene sequences established that there were two genomic regions related to the *WtmsDW* interval of wheat: on chromosome 3 (11.13–12.71 Mb; Nipponbare IRGSP Reference sequence 1.0) and chromosome 7 (12.33–22.39 Mb). The *qHTB3-2* QTL influencing floret fertility responses to heat stress at booting (Zhu et al., 2017) overlapped with the chromosome 3 interval. However, the reported rice QTL interval was relatively large (∼10 Mb; 12.33–22.39 Mb) and only overlapped for ∼400 kb of the ∼1.58 Mb, corresponding to the *WtmsDW* interval.

A grain yield locus responsive to temperature during heading in barley (Romagosa et al., 1999; Malosetti et al., 2004) was located between markers *Rbc2* and *ABG002* on chromosome 2H. This corresponded to 44.36–55.88 Mb on wheat chromosome 2B, which is distal of *WtmsDW*.

### WtmsDW Validation Experiment

**FIGURE 4:**
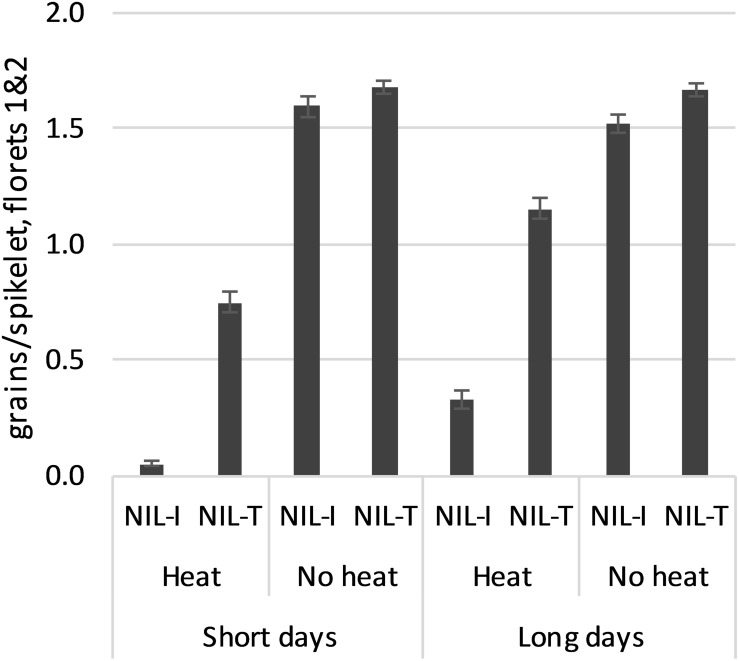
Floret fertility (floret positions 1 and 2; means ± SE) in *WtmsDW* near-isogenic lines, NIL-I (Drysdale allele, intolerant) and NIL-T (Waagan allele, tolerant). All means were significantly different at *p* < 0.001 except among the “No heat” means.

The three NILs selected for each *WtmsDW* allele type behaved similarly (not shown), so were regarded as one for the purposes of data presentation: “NIL-I” with the intolerance allele from Drysdale, and “NIL-T” with the tolerance allele from Waagan, respectively. In the absence of heat stress, fertility was unaffected by day length (9 h vs. 14 h) in either of the NILs (Figure 4). Heat treatment reduced fertility in both NILs, but more so in NIL-I than in NIL-T. Heat stress reduced fertility more in both NILs under short days than under long days, although the relative difference between the NILs remained similar (Figure 4). In other words, while fertility may have responded to day-length, this response was independent of *WtmsDW*. These results validated *WtmsDW* as a floret fertility heat tolerance locus and showed that its effects were not day-length dependent.

DArTSeq^TM^ genomic profiling indicated that the NIL lines were 96% identical, differing only for a segment of chromosome 2B carrying *WtmsDW* (64.23–247.52 Mb) and small segments on chromosomes 1A and 3D which did not carry any of the other reported floret fertility heat-response QTL listed in Table 2. These data further supported the assertion that floret fertility effects observed in the validation experiment were due to *WtmsDW.*

## Discussion

### WtmsDW Significance and Potential Applications

*WtmsDW* is a locus controlling major natural variation for male sterility in response to high temperatures at booting stage in wheat. Variation between the two Australian wheat cultivars Drysdale and Waagan was used to define *WtmsDW*. Drysdale (Hartog^∗^3/Quarrion) was released in 2002, and Waagan (Janz/24IBWSN-244) was released in 2007, both in NSW. The *WtmsDW* alleles present in Drysdale (intolerant) and Waagan (tolerant) were inherited from Hartog and Janz, respectively (marker data not shown). Hartog and Janz have been particularly popular cultivars and (in addition to Drysdale) have been used extensively as parents in Australian wheat breeding. This suggests good potential for *WtmsDW* closest flanking KASP markers *wsnp_JD_c3732_4781170* and *AHW_DW_014* (Supplementary Table 1) to be used in current Australian breeding programs, either to select for heat tolerance or against intolerance. In the validation experiment using *WtmsDW* NIL lines, the tolerance allele offset 44–65% of the losses in grain set due to heat stress otherwise experienced in lines carrying the intolerance allele (Figure 4), suggesting that the use of these KASP markers could lead to substantial yield benefits. However, *WtmsDW* tolerance would need to be evaluated in a number of genetic backgrounds and multiple field environments over a number of years to understand its true value for breeding.

Heat tolerance QTL were previously described in the *WtmsDW* region of chromosome 2B (Figure 3). Mason et al. (2010) identified a QTL in this region for heat response (Heat Susceptibility Index) of grain number per spike, in a cross between the Australian spring cultivar Halberd and the winter wheat Cutter, for heat treatments commencing 10 days after anthesis. However, in our experience, heat treatments applied at this stage in a range of genetic material has not affected grain number (Shirdelmoghanloo et al., 2016, and unpublished data). Zheng et al. (2010) identified a QTL for grain number per square meter that interacted with several environmental variables, including cumulative degree-days over 25°C in the 6 days around meiosis, across 12 field environments, in the winter wheat cross Arche × Récital. Meiosis occurs in stems with an AD around 0–5 cm (Browne et al., 2018), which is around when *WtmsDW* effects were expressed (Supplementary Figure 5). Although further work will be needed to confirm whether these loci are equivalent to *WtmsDW*, these other studies hint that *WtmsDW* selection might be applicable across a wide range of breeding programs.

The Drysdale × Waagan DH population has also been screened for responses to a 3-day heat stress applied at 10 days after anthesis (Shirdelmoghanloo et al., 2016). Loci on chromosomes 3B and 6B influenced the ability to maintain both higher grain weight and flag leaf chlorophyll content under heat, suggesting that grain filling heat tolerance may have a mechanism relating to lower rates of heat-enhanced senescence in both the leaves and grains. By contrast, *WtmsDW* was located on chromosome 2B at a location that was not associated with any chlorophyll QTL effect. Independent genetic control of booting and grain filling stage heat tolerance concurs with the findings of Wardlaw et al. (1989), in which the most tolerant cultivars at the former stage were among the least tolerant at the latter stage. Different developmental stages are sensitive to the floret fertility and grain size responses to heat (booting or anthesis vs. early grain filling, respectively). Therefore, different genes/mechanisms control heat responses of grain weight and number, and breeders need to select the two types of tolerance separately, whether by using markers or phenotyping.

Loci for male sterility that is dependent on particular conditions of day length and/or temperature have been of interest in rice and wheat due to their potential utility in hybrid breeding (reviewed by Fan and Zhang, 2018 and Selva et al., 2020). The *Wtms1*, *TmsBS20T*, and *Wptms2* loci on wheat chromosome 2B are examples. These appeared to be separated from *WtmsDW* on the basis of map position (Figure 3). However, further work will be needed to confirm the separate location of *WtmsDW*, due to potential problems associated with comparing maps from different studies. *Wtms1* and *TmsBS20T* differ from *WtmsDW* in expressing sterility under cold conditions (< 10°C, during spikelet differentiation stage; Xing et al., 2003; Li et al., 2006; Ru et al., 2015) rather than heat, which further supports the proposal that they are different to *WtmsDW*. It was not resolved whether it was long days and/or high temperatures during head development that was required for sterility expression by *Wptms2* (Guo et al., 2006a,b). Sterility sources considered for hybrid breeding are often described as mutant variants (e.g., *Wtms1* and *TmsBS20T*; Xing et al., 2003; Ru et al., 2015) and provide near-total sterility. By contrast, *WtmsDW* is defined by variation between two wheat cultivars. Lines carrying the intolerance allele maintained some fertility under rather severe heat conditions (Figure 4). These initial data suggest *WtmsDW* may not be suitable for use in hybrid wheat breeding.

The DH line WW28450 carried a deletion of the whole of the short arm of chromosome 2B and was heat intolerant. This suggested that tolerance from 2BS derives from positive gene function(s) (as opposed to absence/reduction of a tolerance suppressor), which is missing/reduced in the intolerance allele. However, 2BS carries additional male fertility loci, including *Wptms2* that conditions sterility under high temperatures and/or long days (Figure 3; Guo et al., 2006a), so the implications for *WtmsDW* are not entirely clear.

### Effect of Heat Stress and Heat Stress Timing on Spikelet Fertility

In wheat, floret differentiation usually begins in the middle of the spike and proceeds outwards, and within each spikelet, it begins at the basal most florets and proceeds upwards. The developmental timing differs by up to 5 days across the whole spike (Evans et al., 1972; Tashiro and Wardlaw, 1990b). In the intolerant DH lines carrying the Drysdale *WtmsDW* allele, the various floret positions peaked in heat susceptibility (Figure 2) at times that were broadly consistent with their expected sequence of development. The florets that were the first to develop (i.e., no. 1 and 2 in spikelets from the middle of the spike), peaked in sensitivity at around 5.5 cm AD (Figure 2A), which was 8.6 days (heat) or 11.7 days (control) before anthesis in these florets (based on average timing for 3 and 9 cm AD). In these floret positions and at this AD, anthers were found to be at the meiosis to young microspore stage, depending on the wheat cultivar (Browne et al., 2018; Fernández-Gómez et al., 2020). Our results were in general agreement with other wheat studies that showed a peak in sensitivity to the floret sterility effects of heat stress at 18 days before mid-anthesis (Barber et al., 2017), 6–8 days before anthesis (Prasad and Djanaguiraman, 2014), during meiosis to young microspore stages of pollen development (Saini and Aspinall, 1982), or during premeiotic interphase to late leptotene stage of meiosis (Draeger and Moore, 2017). A similar stage is most sensitive to the fertility responses to drought stress (young microspore stage, at AD 4–6 cm; Ji et al., 2010), suggesting that the same developmental process may be sensitive to both heat and drought. It should be emphasized that our heat treatments did not cover the period 2–3 days before anthesis, which is another stage where wheat can be sensitive to floret fertility effects of heat (Tashiro and Wardlaw, 1990a; Prasad and Djanaguiraman, 2014; Barber et al., 2017).

In floret positions >2, heat stress caused most sterility when it was applied at the latest stages of stem development (6.5 and 9 cm AD), which was consistent with the fact that these florets differentiate after floret positions 1 and 2 (Figure 1A). Unexpectedly, heat exposure at the earliest stage of stem development (1.6 and 3.0 cm AD) enhanced fertility in floret positions >2, particularly in the top third of the spike (Figure 1A). The biological basis for this enhanced fertility is unknown. However, for practical purposes, it had little impact on yield, because even under heat conditions, these floret positions contributed only a small proportion (< 5%) of the grains.

### Floret Fertility Effects Potentially Arising by Escape

In studies of fertility responses to abiotic stresses, AD has often been used as a measure of stem development to time treatments (e.g., Ji et al., 2010; Jagadish et al., 2014). However, any genetic effect that alters the relationship between AD and spike developmental stage could potentially give escape artifacts. The two major dwarfing loci *Rht-B1* and *Rht-D1* appeared to have had such an effect in the Drysdale × Waagan DH population. This population segregated at both loci (Shirdelmoghanloo et al., 2016), and hence included lines that were double-dwarf, semi-dwarf, or tall (carrying dwarfing alleles at both, one or neither of these loci, respectively). Peak sensitivity to the effects of heat on floret sterility occurred at shorter ADs in the double-dwarfs than in the other two classes (Supplementary Figure 4), presumably because double-dwarfs had shorter AD at each of the corresponding pollen developmental stages, owing to their overall shorter AD. Similarly, tall alleles at both *Rht* loci (*Rht-B1a* and *Rht-D1a*) increased the time interval between reaching target AD and anthesis (Supplementary Table 2), indicating that the spike had further to develop at the target AD in the tall genotypes, as compared with the short genotypes. The *Rht-D1* locus also showed QTL effects for floret fertility responses, with the Waagan (tall) allele providing “tolerance” for stems that were heat exposed at the shortest AD (1.6 cm) and the Drysdale (short) allele providing tolerance for stems exposed at the longest AD (9 cm) (Supplementary Table 2). This was consistent with a potential escape mechanism in which spikes were relatively immature at a given AD in plants with potential to be tall (favoring heat exposure prior to the sensitive floret stage), and relatively mature at a given AD in plants with potential to be short (favoring heat exposure after the sensitive stage). This interpretation is supported by the results of Browne et al. (2018) and Fernández-Gómez et al. (2020). They found pollen development stage to be further advanced at a given AD in the semi-dwarf wheats Cranbrook, Young, and Wyalkatchem as compared with the tall cultivars Cadenza and Halberd (*Rht* genotype of these cultivars based on Pearce et al. (2011); and our own unpublished data). For example, an AD of 5.5 cm corresponded to meiosis in the tall cultivars but to post-meiosis (young microspore stage) in the semi-dwarf cultivars, in the most advanced florets of the spike. In the Drysdale × Waagan population, there was a floret fertility response QTL effect on chromosome 4B (*QTL18*), but this was located ∼48 cM from *Rht-B1*. Why there was no fertility response (potential escape) effect observed at *Rht-B1* is unknown.

Other studies found that *Rht* genes influenced responses of floret fertility to heat at booting, although there were inconsistencies, with reported effects ranging from positive to negative, or neutral (Alghabari et al., 2014 and references therein; Barber et al., 2017). The aforementioned issues around staging may at least partly explain these inconsistencies. However, Barber et al. (2017) reported that *Rht-D1* and the flowering time locus *Ppd-D1* affected responses to heat at around anthesis, which is not readily explained by staging artifacts, since anthesis in wheat occurs out of the boot and can usually be observed directly. On this basis, dwarfing/flowering time genes may be capable of genuinely affecting heat tolerance, at least at the anthesis stage.

We established that *WtmsDW* is a genuine heat tolerance locus, as it was not associated with any other trait that indicated the possibility of escape. Awn length at maturity was the only other trait ascribed to this QTL region (Supplementary Table 2). The flowering time locus *Ppd-B1* was mapped 7.2 cM distal of *WtmsDW* using a KASP marker in the *Ppd-B1* gene sequence (Figure 3; Supplementary Figure 1). There was no evidence that the Drysdale × Waagan DH population segregated for functional differences at *Ppd-B1*, at least under these growth conditions, because there were no flowering time QTL effects detected at this location. The closest flowering time effect was located at 26.1 cM (current study) or 5.4 cM (Shirdelmoghanloo et al., 2016) on chromosome 2B (0.7 to 1.5 day effect, with the Drysdale allele conferring lateness), which is well above both *Ppd-B1* (at 74.3 cM) and *WtmsDW* (at 81.5 cM). We have also separated *WtmsDW* from the *Ppd-B1* marker in additional fine mapping work (manuscript in preparation), confirming the independent nature of these loci. There was also no indication that DH lines with the *WtmsDW* tolerance (Waagan) allele had a sensitive stage peaking at just beyond the AD range when treatments were applied (i.e., might have escaped); these lines maintained high levels of fertility for heat treatments across the AD range of 1 to 12 cm (Supplementary Figures 5C,D).

Some fertility *per se* QTL effects were detected at *WtmsDW* under control conditions, but compared with *per se* effects under heat, these were ∼3–10 times weaker in additive effect for comparable floret types and detected less frequently (Supplementary Table 2). The occasional effects observed for control conditions may have been due to a moderately hot day of 27.2°C experienced in the greenhouse during booting. Expression of *WtmsDW* floret fertility effects therefore seemed largely limited to heat stress conditions.

Evidence more or less suggested the remaining four (weaker) fertility response QTL could have been due to escape (Supplementary Table 2). This evidence seemed weakest for *QTL43* and *QTL32*. *QTL43* (43.2 to 67 cM on linkage group 7A2) showed fertility responses and fertility *per se* effects under heat stress and had no developmental effects co-locating with it (Supplementary Table 2). However, height effects mapped nearby (at 24.3 to 33.7 cM in the *QTL28* region). The floret fertility response effect at *QTL32* (66.1 cM on chromosome 1B) also mapped close to floret fertility *per se* effects under heat (at 26.2 cM in the *QTL31* region). However, the *QTL32* region also influenced the degree of awn emergence in tillers on the day that AD on main stems reached 9 cm, implying that it may have affected the relationship between spike stage and AD. Additional work would be needed to validate *QTL43* and *QTL32* fertility tolerance effects, including ruling out potential escape artifacts. However, given the relatively weak effects of these loci (Table 2), they may not be worth the effort.

The fertility tolerance effects at *QTL18* and *QTL39* were much more likely to be due to escape. *QTL18* on chromosome 4B affected multiple aspects of development (Supplementary Table 2; Shirdelmoghanloo et al., 2016). These included flowering time, time to reach target AD, and AD at maturity, which were strong indicators of an escape artifact. Likewise, *QTL39* was associated with plant height.

### Heat-Response QTL for Other Traits

Elevated temperature cannot only accelerate organ expansion but also shorten phases of development and enhance senescence (Parent et al., 2010; Asseng et al., 2011; Hunt et al., 2018). Heat stress decreased AD and plant height at maturity (Figure 1), perhaps by truncating the later stage of growth of these organs. For these two traits, QTL for tolerance genetic effects were observed at six loci, including *Rht-B1* and *Rht-D1* (Table 3). However (except at *QTL34*), the tolerance alleles were associated with lower *per se* value under control (and heat), consistent with escape, i.e., genotypes with potential for shorter height or AD had completed more of their potential growth at the time of reaching the target AD for heat treatment, and hence had less opportunity to be affected by the heat stress. *QTL34* on chromosome 2A showed a tolerance effect for height but no *per se* height effect, raising the possibility that it may have been a genuine tolerance effect.

Heat stress enhanced spike length at maturity by increasing rachis internode length but decreased awn length (as measured from the last glume). *QTL9* and *QTL29* affected heat responses of spike length and awn length, respectively, with positive alleles for heat tolerance effects (conditioning greater positive response and smaller negative response, respectively) also being positive for greater length *per se* of these organs. Elevated temperature therefore appeared to enhance further the tendency of these alleles to promote organ length, perhaps because these organs would have been in a phase of rapid growth during the treatments (based on comparing target ADs with the data of Browne et al., 2018). The *QTL41* awn length response effect was not associated with any other trait but was relatively weak.

Typically, a small number of spikelets at the bottom of the wheat spike are relatively underdeveloped and do not set grain. Five loci (*QTL5.2*, *18*, *25*, and both *Rht* loci) affected the tendency of heat treatment to convert such spikelets to a “developed” state (defined in this study as awns longer than half the length of those from the middle of the spike; Table 3). Except at *QTL5.2*, the alleles that favored this conversion conferred higher *per se* numbers of underdeveloped spikelets, probably reflecting the fact that genotypes with the potential to have higher numbers of underdeveloped spikelets had more such spikelets to convert. These loci also affected plant height and/or AD at maturity, with tall alleles being associated with a lower *per se* number of underdeveloped spikelets, suggesting a physiological link between plant height and the ability of these basal spikelets to develop further.

*QTL29* affected the time interval from target AD to anthesis, without affecting AD at maturity or flowering time. Heat magnified the genetic effect, as the Drysdale allele was positive for both the *per se* trait in control and heat tolerance effect. It was surprising that *QTL29* showed no fertility response (escape) effect, as the findings implied it affected the relationship between spike developmental stage and AD.

*QTL30* on chromosome 1A and *QTL33* on chromosome 1B only affected heat responses of flowering time. These loci may represent functionally orthologous genes, since these two chromosomes are related (orthologous), and the corresponding positions of the loci were only 25–30 cM apart on the respective maps, based on BLAST searches with the peak markers (data not shown).

## Conclusion

The *WtmsDW* locus on the short arm of wheat chromosome 2B defines a major natural variation for responses of male fertility to heat stress at booting, suggesting *WtmsDW*-linked markers may have substantial value in heat tolerance breeding. In lines carrying the *WtmsDW* intolerance allele, peaks in sensitivity of the various different floret positions were defined in relation to auricle distance. Mapping of height and flowering time traits proved very useful in identifying when floret fertility response QTL were likely to be due to escape artifacts. These insights should be valuable for guiding future efforts to screen for booting stage heat tolerance in wheat.

## Author’s Note

This article is dedicated to the memory of Colin F. Jenner.

## Data Availability Statement

’The original contributions presented in the study are included in the article/Supplementary Material, further inquiries can be directed to the corresponding authors.

## Author Contributions

ME and NC conceived the study and drafted the manuscript. JT generated the experimental design. IM-S conducted the phenotypic analyses. ME conducted the QTL analyses. ME, IL, LE, and NC performed the experimental work. All authors read and revised the manuscript.

## Conflict of Interest

The authors declare that the research was conducted in the absence of any commercial or financial relationships that could be construed as a potential conflict of interest.

## Footnotes

1 https://wheat.pw.usda.gov/GG3/

2 http://gramene.org/

3 https://www.ncbi.nlm.nih.gov/

4 https://www.diversityarrays.com/

5 http://rice.plantbiology.msu.edu/analyses_search_blast.shtml
