# Correction: The *WtmsDW* locus on wheat chromosome 2B controls major natural variation for floret sterility responses to heat stress at booting stage

**DOI:** 10.3389/fpls.2025.1723691

**Published:** 2025-11-27

**Authors:** Million F. Erena, Iman Lohraseb, Isabel Munoz-Santa, Julian D. Taylor, Livinus C. Emebiri, Nicholas C. Collins

**Affiliations:** 1 School of Agriculture Food and Wine, The University of Adelaide, Adelaide, SA, Australia; 2 Department of Statistics and Operations Research, University of Valencia, Valencia, Spain; 3 Graham Centre for Agricultural Innovation, Charles Sturt University, Wagga Wagga, NSW, Australia; 4 New South Wales Department of Primary Industries, Wagga, Wagga, NSW, Australia

**Keywords:** wheat, heat tolerance, male sterility, floret sterility, auricle distance, QTL

In the published article, there were errors in the **Results** and **Discussion** sections, as well as **Data Sheet 1**. These errors became apparent when the seed stock of the doubled-haploid (DH) line WW28450 was subsequently found to be a mixture of two DH lines. These comprised one line that was Drysdale-type non-recombinant for chromosome arm 2BS, and a recombinant line that was Waagan-type for the 2BS *WtmsDW* interval and Drysdale-type proximally. The authors wrongly interpreted the marker calls to indicate that WW28450 had a deletion of the *WtmsDW* interval when in fact there was no such deletion.

The following text, located in **Results**, *Further Mapping in the WtmsDW Region and Relationships to Other Fertility Loci*, Paragraph two, has been removed:

“The DH line WW28450 carried a spontaneous deletion on chromosome 2B from 104.24 Mb upwards (**Supplementary Figure 1**), essentially covering the whole of the short arm, including the *WtmsDW* locus (**Figure 3**). This line was phenotypically intolerant to heat stress but fully fertile under control conditions (**Supplementary Figure 1**).”

The following text located in **Discussion**, *WtmsDW Significance and Potential Applications*, paragraph five, has been removed:

“The DH line WW28450 carried a deletion of the whole of the short arm of chromosome 2B and was heat intolerant. This suggested that tolerance from 2BS derives from positive gene function(s) (as opposed to absence/reduction of a tolerance suppressor), which is missing/reduced in the intolerance allele. However, 2BS carries additional male fertility loci, including *Wptms2* that conditions sterility under high temperatures and/or long days (**Figure 3**; Guo et al., 2006a), so the implications for *WtmsDW* are not entirely clear.”

The following text, located in **Data Sheet 1**, **Supplementary Figure 1***, Drysdale × Waagan DH mapping line information* has also been removed: “Line WW28450 (column CK) was inferred to be a deletion mutant from 104.24 Mb upwards based on 9K SNP marker calls”.

The original version of the article has been updated.

