# Supplementary material for: The WtmsDW Locus on Wheat Chromosome 2B Controls Major Natural Variation for Floret Sterility Responses to Heat Stress at Booting Stage
Source: Front Plant Sci. 2021 Mar 29;12:635397. doi: 10.3389/fpls.2021.635397 (PMC8040955; doi:10.3389/fpls.2021.635397)
Supplement: Supplementary file 2 [file Data_Sheet_2.pdf]

Erena et al. *WtmsDW* heat tolerance locus

Supplemental Figures 2–5

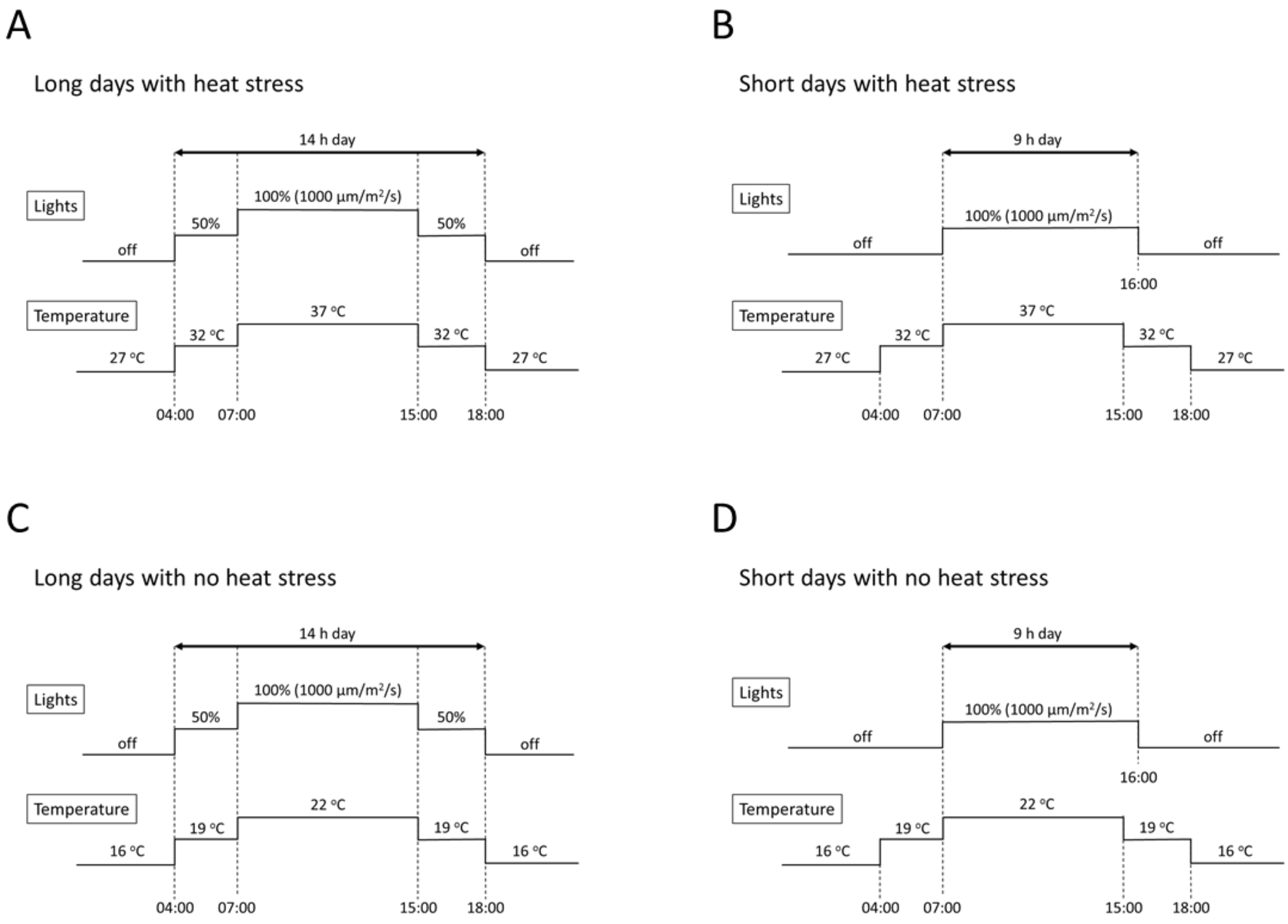

Supplemental Figure 2 Temperature and light treatment regimes: Long days with heat stress (A), short days with heat stress (B), long days with no heat stress (C) and short days with no heat stress (D). The program in A was the one used for the DH QTL experiment. All four programs were used in the *WtmsDW*-validation experiment.

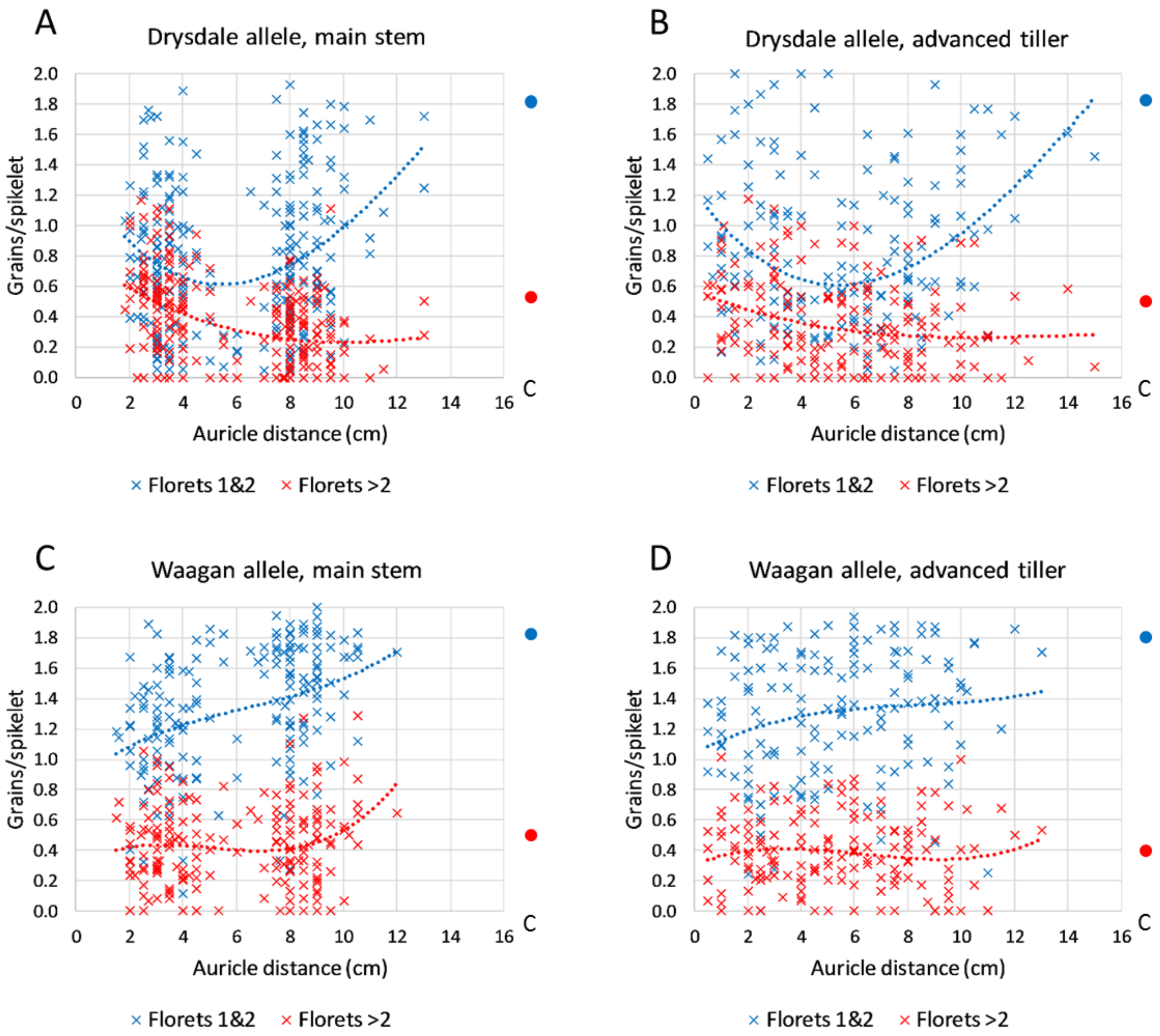

Supplemental Figure 3 Grain set on individual stems of Drysdale × Waagan DH mapping lines plotted against auricle distance on the stem at the beginning of the 3 d heat treatment. Data for lines carrying contrasting alleles at *WtmsDW* locus are shown: Drysdale intolerance allele (A,B), and Waagan tolerance allele (C,D). Data are for main stem (A,C) and the most advanced tiller (B,D). Data for floret positions 1&2 and >2 on the spikelets were plotted separately and used to fit regression curves (polynomial order-3). Average fertility in control plants (C) are represented by the dots to the right of each plot. Average auricle distance in control plants at maturity was 18.9 cm for main stems and 18.5 cm for tillers.

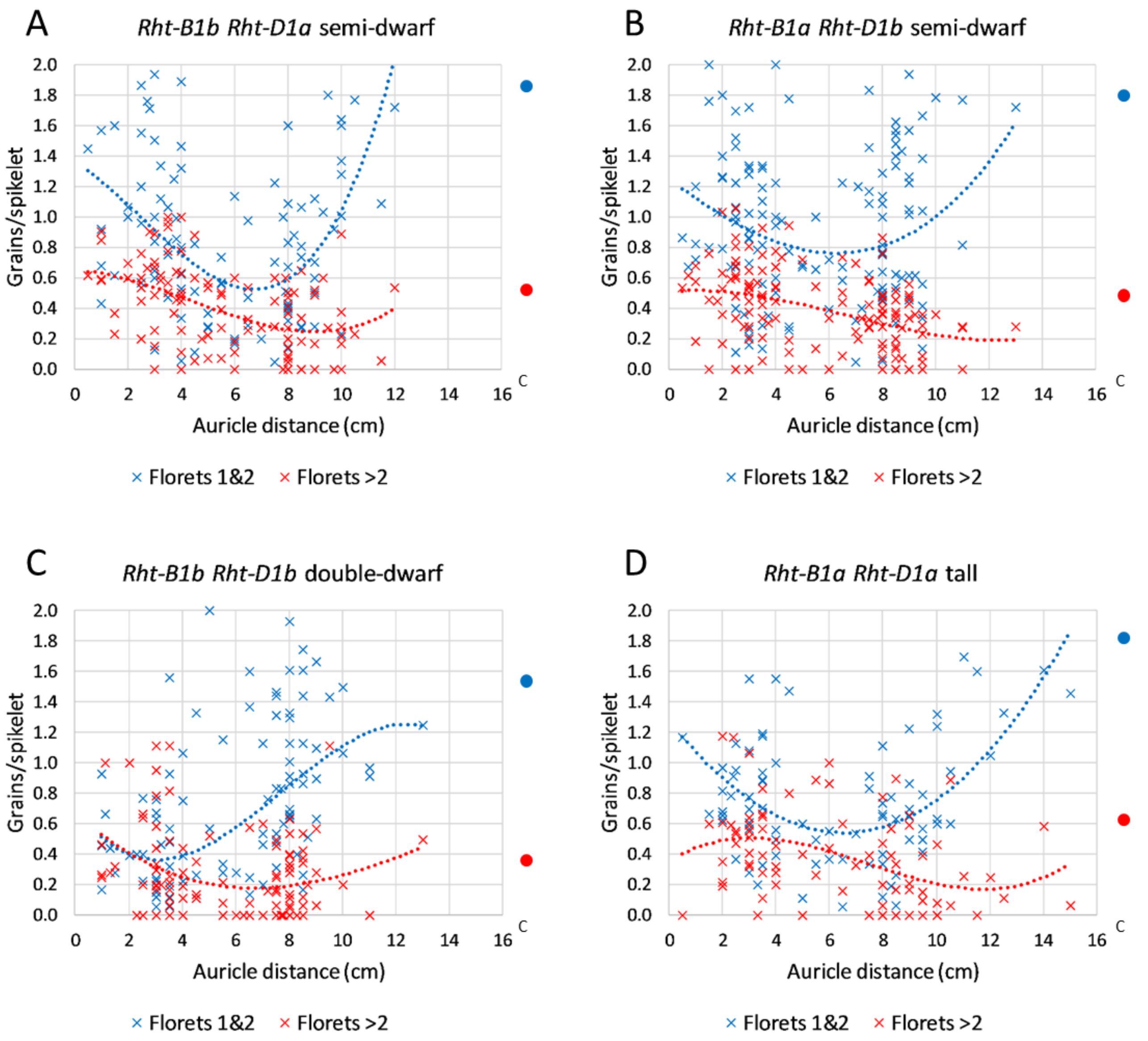

Supplemental Figure 4 Grain set on individual stems of Drysdale × Waagan DH mapping lines carrying the intolerance (Drysdale) allele of *WtmsDW*, plotted against auricle distance on the stem at the beginning of the 3 d heat treatment. Data for main stems and advanced tillers were combined. Data for lines carrying different combinations of the *Rht-B1* and *Rht-D1* dwarfing loci are shown: semi-dwarf types *Rht-B1b Rht-D1a* (A) and *Rht-B1a Rht-D1b* (B), *Rht-B1b Rht-D1b* double-dwarfs (C) and *Rht-B1a Rht-D1a* talls (D). Data for floret positions 1&2 and >2 in the spikelets were plotted separately and used to fit regression curves (polynomial order-3). Average fertility in control plants (C) are represented by the dots to the right of the plots. Average auricle distance in control plants at maturity was 19.3, 19.0, 12.3 and 25.5 cm for A, B, C and D, respectively.

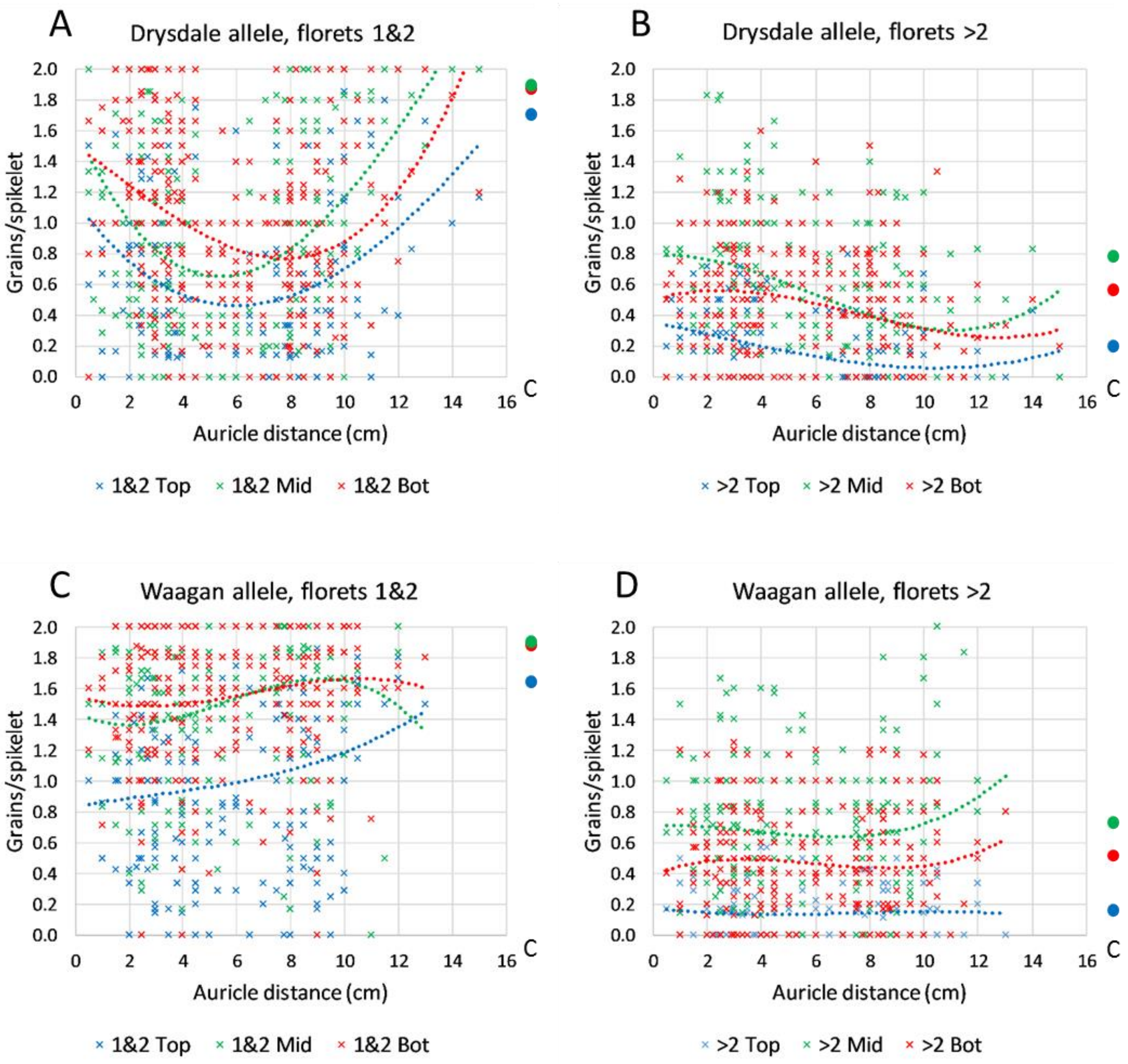

Supplemental Figure 5 Grain set on individual stems of Drysdale × Waagan DH mapping lines, plotted against auricle distance on the stem at the beginning of the 3 d heat treatment. Data for main stems and advanced tillers were combined and double-dwarfs were excluded. Lines carrying the *WtmsDW* Drysdale intolerance allele (A,B) and Waagan tolerance allele (C,D) are shown separately, as are data for floret positions 1&2 (A,C) and >2 (B,D) on the spikelets. Grain set in the top, middle and bottom third of the spike were used to fit regression curves (polynomial order-3). Average fertility in control plants (C) are represented by the dots to the right of the plots. Average auricle distance in control plants at maturity was 20.8 cm. A and B represent the same data as Figure 2.
